# DJ-1 Protects Pancreatic Beta Cells from Cytokine- and Streptozotocin-Mediated Cell Death

**DOI:** 10.1371/journal.pone.0138535

**Published:** 2015-09-30

**Authors:** Deepak Jain, Gesine Weber, Daniel Eberhard, Amir E. Mehana, Jan Eglinger, Alena Welters, Barbara Bartosinska, Kay Jeruschke, Jürgen Weiss, Günter Päth, Hiroyoshi Ariga, Jochen Seufert, Eckhard Lammert

**Affiliations:** 1 Institute of Metabolic Physiology, Heinrich Heine University, Düsseldorf, Germany; 2 Institute for Beta Cell Biology, German Diabetes Center at Heinrich Heine University, Leibniz Center for Diabetes Research, Düsseldorf, Germany; 3 German Center for Diabetes Research (DZD e.V.), Düsseldorf Partner Institute, Düsseldorf, Germany; 4 Division of Endocrinology and Diabetology, Department of Internal Medicine II, University Hospital of Freiburg, Freiburg, Germany; 5 Department of Zoology, Faculty of Science, Suez Canal University, Ismailia, Egypt; 6 Department of General Pediatrics, Neonatology and Pediatric Cardiology, University Children’s Hospital Düsseldorf, Düsseldorf, Germany; 7 Institute of Clinical Biochemistry and Pathobiochemistry, German Diabetes Center at Heinrich Heine University, Leibniz Center for Diabetes Research, Düsseldorf, Germany; 8 Graduate School of Pharmaceutical Sciences, Hokkaido University, Kita-Ku, N12 W6, Sapporo, Japan; Joslin Diabetes Center, Harvard Medical School, UNITED STATES

## Abstract

A hallmark feature of type 1 and type 2 diabetes mellitus is the progressive dysfunction and loss of insulin-producing pancreatic beta cells, and inflammatory cytokines are known to trigger beta cell death. Here we asked whether the anti-oxidant protein DJ-1 encoded by the Parkinson’s disease gene *PARK7* protects islet cells from cytokine- and streptozotocin-mediated cell death. Wild type and DJ-1 knockout mice (KO) were treated with multiple low doses of streptozotocin (MLDS) to induce inflammatory beta cell stress and cell death. Subsequently, glucose tolerance tests were performed, and plasma insulin as well as fasting and random blood glucose concentrations were monitored. Mitochondrial morphology and number of insulin granules were quantified in beta cells. Moreover, islet cell damage was determined *in vitro* after streptozotocin and cytokine treatment of isolated wild type and DJ-1 KO islets using calcein AM/ethidium homodimer-1 staining and TUNEL staining. Compared to wild type mice, DJ-1 KO mice became diabetic following MLDS treatment. Insulin concentrations were substantially reduced, and fasting blood glucose concentrations were significantly higher in MLDS-treated DJ-1 KO mice compared to equally treated wild type mice. Rates of beta cell apoptosis upon MLDS treatment were twofold higher in DJ-1 KO mice compared to wild type mice, and *in vitro* inflammatory cytokines led to twice as much beta cell death in pancreatic islets from DJ-1 KO mice versus those of wild type mice. In conclusion, this study identified the anti-oxidant protein DJ-1 as being capable of protecting pancreatic islet cells from cell death induced by an inflammatory and cytotoxic setting.

## Introduction

Both, type 1 and type 2 diabetes mellitus (T1DM and T2DM) are associated with a progressive dysfunction and loss of beta cells in pancreatic islets (or islets of Langerhans) [1–3]. In T1DM, beta cells are targeted by infiltrating immune cells which release pro-inflammatory cytokines such as interleukin-1 beta (IL-1β), interferon-gamma (IFN-γ) and tumour necrosis factor-alpha (TNF-α) known to trigger islet cell death [1, 4, 5]. In contrast, in T2DM, beta cells deteriorate much slower due to accumulating effects resulting from gluco- and lipotoxicity, oxidative and endoplasmatic reticulum stress caused by insulin resistance in the first place [6]. Interestingly, humans with established T2DM also show increased circulating pro-inflammatory cytokine levels and display low-grade islet inflammation suggesting that an inflammatory stress contributes to beta cell dysfunction and death in T2DM [4, 7–9].

We and others have recently analysed in beta cells the role of the anti-oxidant protein DJ-1 that is highly expressed in mouse and human pancreatic islets [10–12]. DJ-1 expression in pancreatic islets is up-regulated by hyperglycemia, increases in human islets with an increasing age of the donor, is decreased in human T2DM islets, and helps to protect the integrity and function of islet mitochondria from oxidative stress possibly ensuring physiologic glucose-stimulated insulin secretion during aging and under conditions of insulin resistance [10, 11]. Moreover, and in analogy to the protective effect of DJ-1 in neurons [13, 14], DJ-1 is probably required in pancreatic islets to protect beta cells from oxidative stress, since beta cells express low amounts of other anti-oxidant proteins [10, 12, 15, 16].

Since beta cells and neurons share many common features, we hypothesize that DJ-1 protein expression could also participate in the protection from cytokine-induced diabetogenic insults especially as DJ-1 has also been suggested to be protective against oxidative stress mediated apoptotic death [17, 18]. In this report, we investigated the islet cell protective effects of DJ-1 in streptozotocin-mediated islet cell death and cytokine-induced beta cell apoptosis *in vitro*, and in the multiple low doses streptozotocin (MLDS) model causing insulitis *in vivo* [19, 20]. We show that in the absence of DJ-1, islet cells display a lower resistance to inflammation- and streptozotocin-induced cell death and loose their cellular integrity accompanied with a severely impaired glucose tolerance.

## Materials and Methods

### Animals

Control (C57BL/6J) and DJ-1 KO (B6.Cg-*Park7* ^*tm1Shn*^/J) mice were purchased from Jackson Laboratory [21], and fed with standard laboratory chow diet (Sniff GmbH, Germany), and drinking water *ad libitum*. 12–13 weeks-old male C57BL/6J mice were purchased from Janvier (Saint Berthevin, France), and used for pancreatic islet isolation as previously described [22]. The local animal ethics committees of the Regierungspräsidium Freiburg, Baden Württemberg, Germany and the Landesamt für Natur, Umwelt und Verbraucherschutz, North Rhine-Westphalia, Germany approved all experiments.

### In vivo treatments

Streptozotocin (STZ, Sigma-Aldrich) was dissolved in 10 mM citrate buffer, pH 4.5, and 40 mg STZ/kg body weight were immediately injected intraperitoneally (i.p.) into mice for five consecutive days [20]. Glucose tolerance tests were carried out by i.p. injections of 1 g glucose/kg body weight into mice after overnight starvation. Plasma insulin concentrations were measured in 14–16 weeks-old male DJ-1 KO and wild type mice after STZ treatment using an ultrasensitive rat insulin ELISA (Crystal Chem, Chicago, IL, USA).

### Live-Dead islet imaging following STZ treatment

Isolated pancreatic islets from male 12–13 weeks-old DJ-1 KO and control mice were incubated for 24 hours in CMRL medium (Life Technologies) containing 0.5 mM STZ in 10 mM citrate buffer (pH 4.5). Following treatment, whole islets were stained with 2.5 μM calcein AM (living cells, Live/Dead Viability/Cytotoxicity Assay Kit, Life Technologies), 4 μM ethidium homodimer-1 (dead cells, Live/Dead Viability/Cytotoxicity Assay Kit, Molecular Probes by Life Technologies) and Hoechst 33342 (cell nuclei, Life Technologies), incubated for 1 h at 37°C and visualized via live-cell imaging using a laser scanning microscopy (LSM) 710 confocal microscope (Zeiss). The relative number of dead cells was quantified by calculating the ratio of the dead cell areas (ethidium homodimer-1 positive areas) to the total cell areas (Hoechst 33342 positive areas), given as percentage using Fiji/ImageJ software.

### Cytokine treatment/TUNEL staining

Isolated pancreatic islets from control as well as DJ-1 KO mice were treated for 24 h with a cytokine mixture, i.e. 50 U/ml IL-1β (R&D Systems) plus 1,000 U/ml IFN-γ (Biosciences) and 1,000 U/ml TNF-α (R&D Systems). The number of apoptotic cells was determined by terminal deoxynucleotidyl transferase-mediated 2'-deoxyuridine 5'-triphosphate nick-end labelling (TUNEL). Briefly after cytokine treatment, isolated islets were washed with PBS, fixed in Bouin’s solution (Sigma) for 15 minutes, washed again with PBS and embedded in OCT medium. 12 μm cryo-sections were used to determine cell apoptosis by incubating them first with 20 μg/μl proteinase K (Applichem) diluted in 10 mM Tris/HCl (pH 7.5, 1:500 dilution) for 10 minutes at 37°C, followed by TUNEL technique according to the manufacturer's instructions (In Situ Cell Death Detection Kit, TMR red; Roche Diagnostics). For the distinction of apoptotic beta cells versus apoptotic alpha cells, the islet sections were first fixed with 4% paraformaldehyde for 15 minutes, followed by an incubation in 50 mM ammonium chloride in 0.2% Triton X 100 for 5 minutes, and at last stained for insulin (1:250 dilution, DAKO) and glucagon (1:200 dilution, Santa Cruz Biotechnology). Secondary antibodies were conjugated to Alexa Fluor 488 (Molecular Probes) and to Cy 5 (Dianova). DAPI (Sigma) was used to stain cell nuclei. Pancreata from STZ treated WT and DJ-1 KO mice were isolated, embedded, and sectioned. The sections were analysed for apoptosis using TUNEL technique and for insulin by immunofluorescence as described above. In order to identify apoptotic beta cells, the number of TUNEL positive cell nuclei within an insulin positive area was manually counted and compared to the total beta cell number manually counted as DAPI and insulin positive staining, given as percentage.

### Measurement of beta cell area and immunofluorescence

After four weeks of MLDS treatment, pancreata of wild type and DJ-1 KO mice were isolated and fixed in 4% paraformaldehyde for 24 h. Evenly spaced 10 μm sections were used to determine the beta cell area by staining them for insulin using the polyclonal guinea pig anti-insulin antibody (DAKO). As secondary antibody we used goat anti-guinea pig conjugated with Alexa-Fluor-555 (Molecular Probes). DAPI (Sigma) was used to stain cell nuclei. Relative insulin-positive area was determined by quantification of the cross-sectional insulin-positive area divided by the cross-sectional area of the whole pancreatic section (nuclei area) and presented as percentage of control. Co-staining of DJ-1 and insulin in pancreatic sections was performed using rabbit anti-DJ-1 (1:100, [23]) and guinea pig anti-Insulin (1:300 dilution, DAKO).

### Electron microscopy

Islets were fixed for 1 h at room temperature by immersion in 2.5% glutaraldehyde in PBS buffer at pH 7.4, postfixed in 2% osmium tetroxide in 0.19 M sodium cacodylate buffer, pH 7.4, for 30 minutes, and subsequently stained with 2% uranyl acetate in maleate buffer, pH 4.6. The specimens were dehydrated in graded ethanols and embedded in epoxy resin [24]. Ultrathin sections were picked up onto Formvar-carbon-coated grids, stained with lead citrate, and viewed in a transmission electron microscope (TEM 910; Zeiss Elektronenmikroskopie, Oberkochen, Germany).

### Quantification of secretory vesicles and mitochondria in electron micrographs

The histograms of electron micrograph (EM) pictures were normalized to a grey level intensity mean of 128 and a standard deviation of 40. Subsequently, a classifier was trained on a subset of images using the Trainable Weka Segmentation plugin included in Fiji/ImageJ [25], defining four segmentation classes: nuclei, mitochondria, secretory vesicles, and cytoplasm (background). The classifier was then applied to all images, resulting in a probability map for each class and every image. The mitochondrial area and number of secretory vesicles were then quantified using automated threshold of the respective probability map image and particle analysis of Fiji/ImageJ.

### cDNA synthesis and quantitative real time PCR

Total RNA was purified from isolated islets of wild type and DJ-1 KO mice using the RNeasy Kit (Qiagen, Hilden, Germany). cDNA was synthesized using M-MLV RT (Promega, Madison, USA). Real time PCR was performed on a Mx3000P machine (Agilent Technologies/Stratagene) using Brilliant III SYBR Green QPCR Mastermix (Agilent Technologies). Expression changes relative to the housekeeping gene hypoxanthine-guanine phosphoribosyltransferase (HPRT) were calculated according to Schmittgen et al. [26]. The following primers were used:

DJ-1, 5´-AGCCGGGATCAAAGTCACTG-3´; 5´-GGTCCCTGCGTTTTTGCATC-3´; HPRT, 5´-GCTGGTGAAAAGGACCT-3´; 5´-CACAGGACTAGAACACCT-3´; CD68, 5´-ATCCCCACCTGTCTCTCTCA-3´; 5´-ACCGCCATGTAGTCCAGGTA-3´; IL-1β, 5´-GCAGCAGCACATCAACAAG-3´; 5´-GTTCATCTCGGAGCCTGTAG-3´; TNF-α 5´-TCTTCTCATTCCTGCTTGTGG; 5´-GGTCTGGGCCATAGAACTGA-3´.

### Statistical analysis

Unpaired, two-tailed Student’s t-test (unequal variances) or one- or two-way analysis of variance (ANOVA) followed by Tukey´s multiple comparison test was used to test statistical significance. Statistical analysis was performed using Microsoft Excel or Graphpad PRISM.

## Results

### DJ-1 contributes to protection from MLDS-induced diabetes

We first tested the possibility that DJ-1 influences the metabolic phenotype induced by MLDS treatment and investigated DJ-1 KO versus wild type mice. In wild type mouse islets, DJ-1 is broadly expressed, whereas islets of DJ-1 KO mice have significantly reduced levels of DJ-1 as observed by real time RT-PCR and immunohistochemistry (S1 Fig), indicating an efficient depletion of DJ-1.

**Fig 1:**
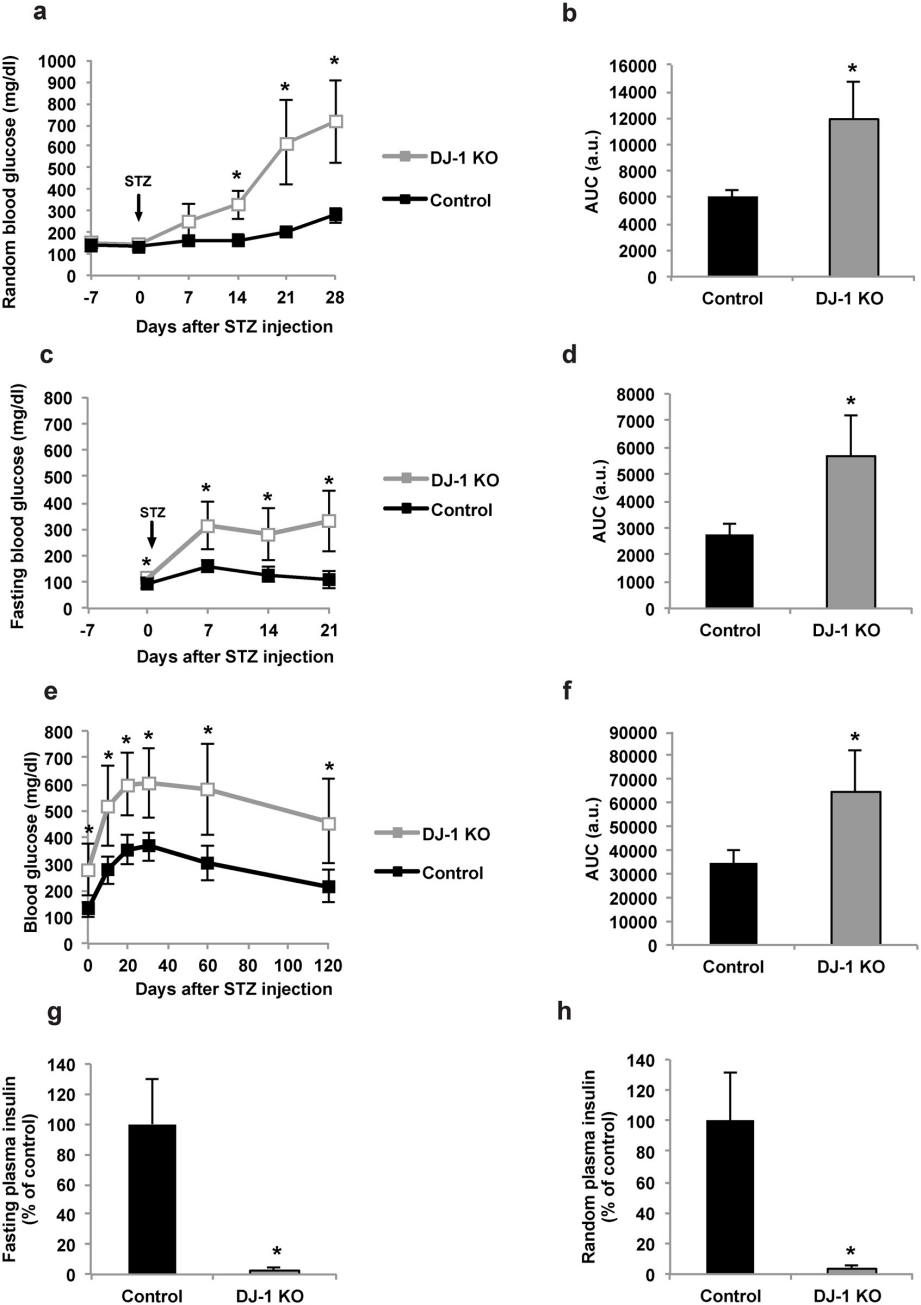
MLDS treatment induces a diabetic phenotype in the absence of DJ-1. Male control and DJ-1 KO mice at 12–13 weeks of age were treated with 40 mg STZ/kg body weight on five consecutive days. Blood glucose concentrations, glucose tolerance, and plasma insulin concentrations were determined. **(a,c)** Random **(a)** and fasting **(c)** blood glucose concentrations in control (black squares) and DJ-1 KO mice (grey squares). n = 6–8 mice per experimental group. (**b**,**d**) Corresponding areas under the curve (AUC) to (**a**,**c**) are shown for control (black columns) and DJ-1 KO (grey columns) mice each. **(e,f)** Glucose tolerance test **(e)** and its corresponding AUC **(f)** in 14–16 weeks-old control (black squares and black column) and DJ-1 KO mice (grey squares and grey column). The glucose tolerance test was performed after intraperitoneal administration of glucose (1 g/kg body weight). n = 8 mice per experimental group. **(g,h)** Relative fasting **(g)** and non-fasting **(h)** plasma insulin concentrations in 14–16 weeks-old control (black columns) and DJ-1 KO mice (grey columns) normalised to controls. n = 8 mice per experimental group in (**g**) and n = 5 in (**h**). *p<0.05 (Student’s t-test in **b, d, f-h**. Student’s t-test with Holm-Bonferroni correction in **a, c, e**). All values are means ± SD.

DJ-1 KO and wild type mice received 40 mg STZ/kg body weight on five consecutive days. The body weight, random and fasting blood glucose concentrations as well as glucose tolerance were monitored. In both groups (wild type and DJ-1 KO mice), MLDS treatment increased random blood glucose concentrations over the course of 28 days (Fig 1a). However, the effect was significantly more pronounced in mice lacking DJ-1 (Fig 1a and 1b). In addition, compared to MLDS-treated wild type mice, DJ-1 KO led to higher fasting blood glucose concentrations (Fig 1c and 1d), and glucose tolerance was impaired in DJ-1 KO mice (Fig 1e and 1f). DJ-1 KO mice also lost weight in the weeks following the STZ injections, whereas the body weight of MLDS-treated wild type mice remained unchanged (S2 Fig). Since both the fasting and random plasma insulin concentrations were significantly lower in DJ-1 KO mice compared to MLDS-treated wild type mice (Fig 1g and 1h), the data suggest that DJ-1 helps to protect pancreatic islets from cell death induced by the MLDS treatment.

### DJ-1 helps to prevent beta cell apoptosis and maintain beta cell area after MLDS treatment

**Fig 2:**
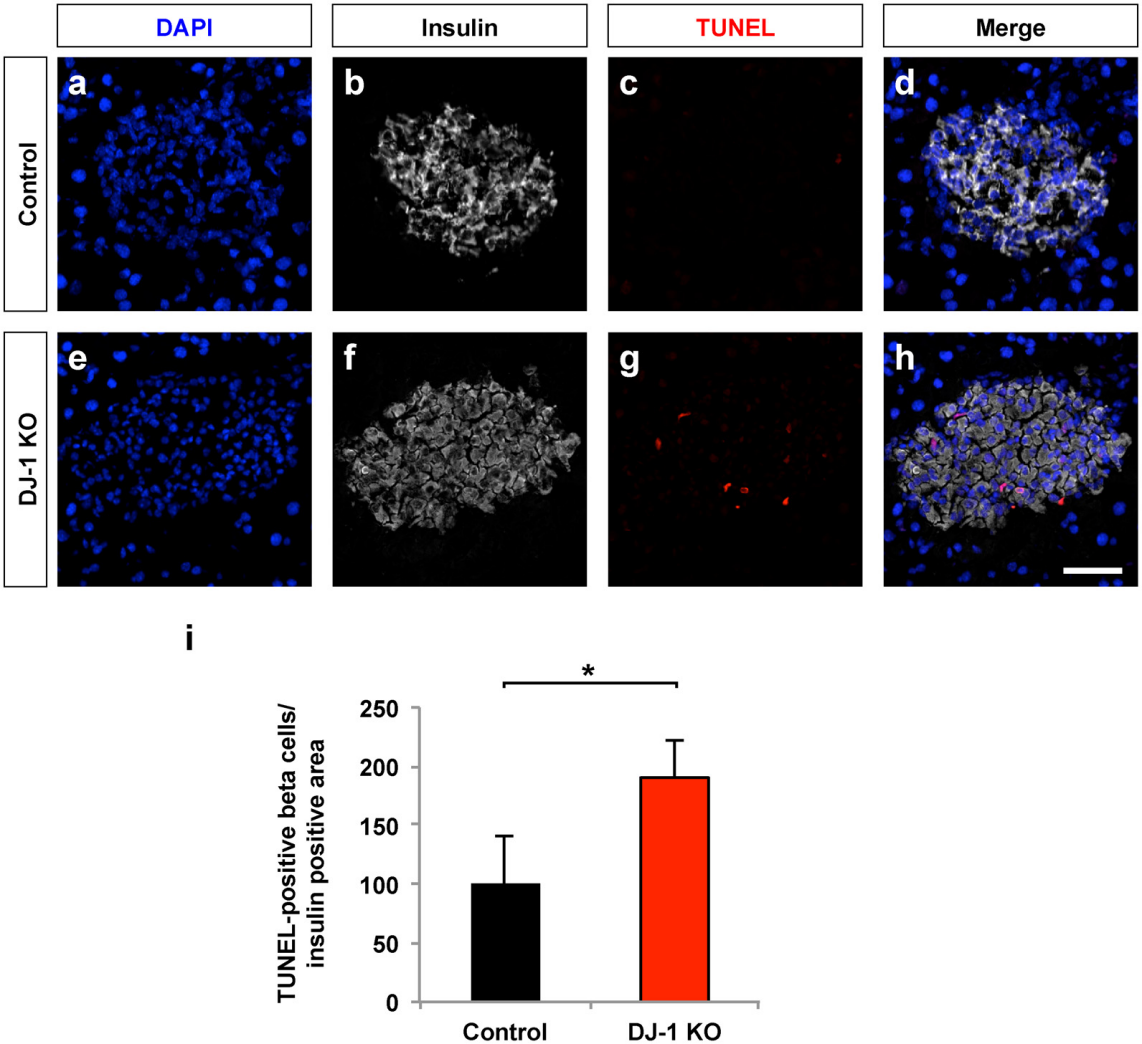
DJ-1 is required to lower the rate of apoptosis upon MLDS treatment. (**a-h**) LSM images of pancreatic islets in sections of pancreata from male control (**a-d**) and DJ-1 KO mice (**e-h**) stained for cell nuclei (DAPI) (**a,e**), insulin (**b,f**) and TUNEL positive nuclei (**c,g**). Merged images (**d,h**) are also shown. Scale bar, 25 μm. (**i**) Quantification of the relative amount of TUNEL positive nuclei in pancreatic islets of DJ-1 KO and control mice, calculated as total number of TUNEL positive apoptotic nuclei per insulin positive area expressed as percentage of control. n = 3 pancreata per experimental group. *p<0.05 (Student’s t-test). Data are expressed as means ± SD.

**Fig 3:**
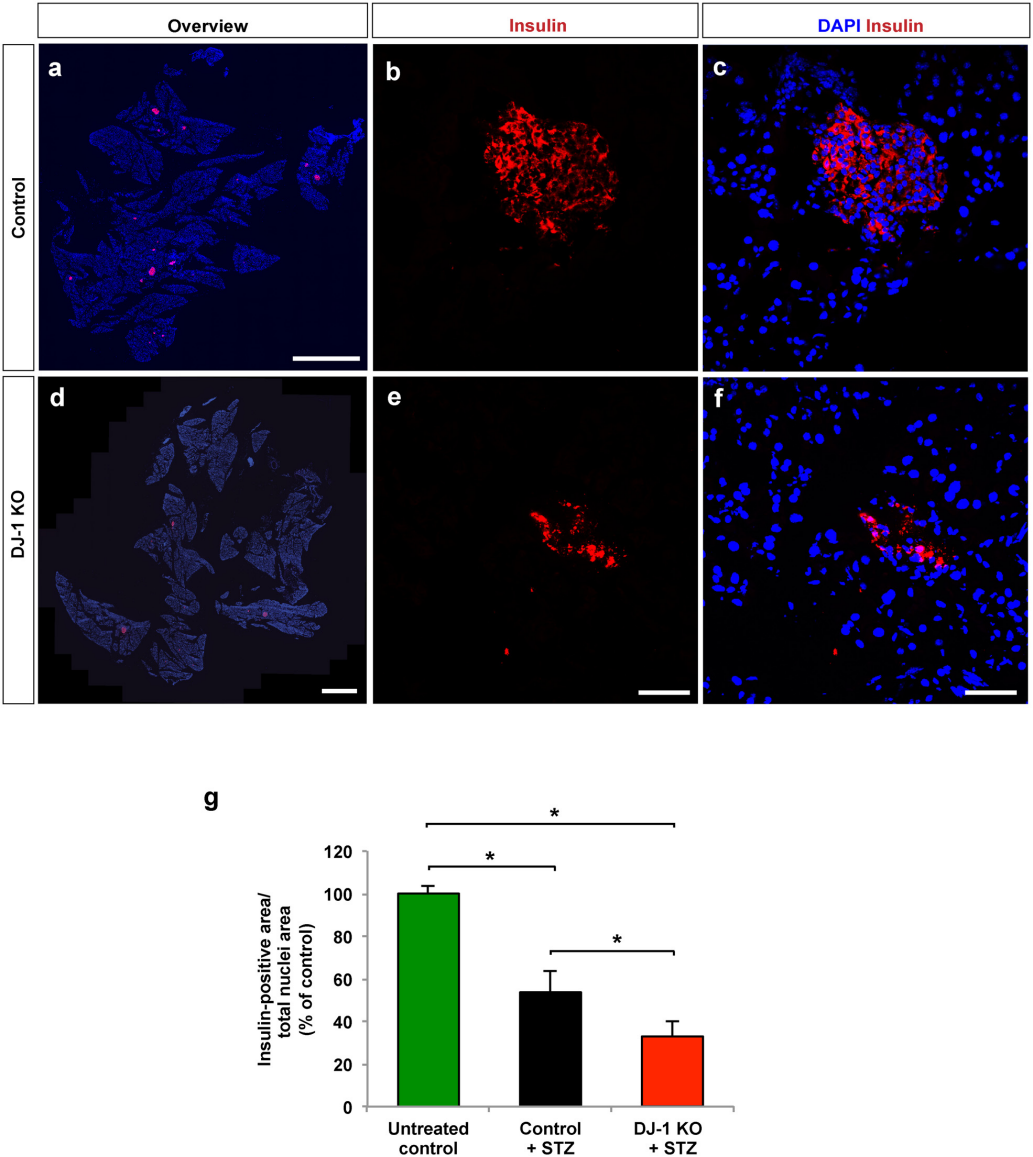
DJ-1 is required to reduce loss of beta cell mass following treatment with MLDS. **(a,d)** Representative fluorescence microscopy images of pancreatic sections from 16 weeks-old male control and DJ-1 KO mice following MLDS treatment, stained for cell nuclei (DAPI) and beta cells (insulin). Scale bars, 0.5 mm. (**b**,**c**,**e**,**f**) LSM images of pancreatic islets in sections of pancreata from male control **(b**,**c)** and DJ-1 KO mice **(e-f)** after MLDS treatment, stained for cell nuclei (DAPI) and beta cells (insulin) **(b,e).** Merged images **(c,f)** are also shown. Scale bars, 50 μm. **(g)** Morphometric analyses of relative beta cell area from DJ-1 KO and control mice calculated as insulin-positive area per total nuclei area of evenly spaced pancreatic sections. n = 3 mouse pancreata per experimental group. Beta cell area was quantified after four weeks of STZ treatment. For comparison, untreated control/wild type mice without STZ treatment were included. *p<0.05 (One-way ANOVA followed by Tukey´s multiple comparison test). Data are expressed as means ± S.D.

MLDS treatment was shown to induce beta cell death, primarily apoptosis. Therefore, the extent of beta cell apoptosis was determined using pancreatic sections obtained from DJ-1 KO and wild type mice treated with MLDS. In DJ-1 KO mice, significantly more apoptotic pancreatic beta cells were found compared to equally treated wild type mice, suggesting that DJ-1 is required to reduce the extent of beta cell apoptosis following treatment with MLDS (Fig 2). Consistent with this notion, MLDS-treated DJ-1 KO mice had a stronger reduction in the beta cell area compared to MLDS-treated wild type mice (Fig 3), indicating that DJ-1 helps to preserve the beta cell mass upon MLDS treatment.

### DJ-1 is required for maintaining mitochondrial morphology and the number of insulin secretory granules after MLDS treatment

**Fig 4:**
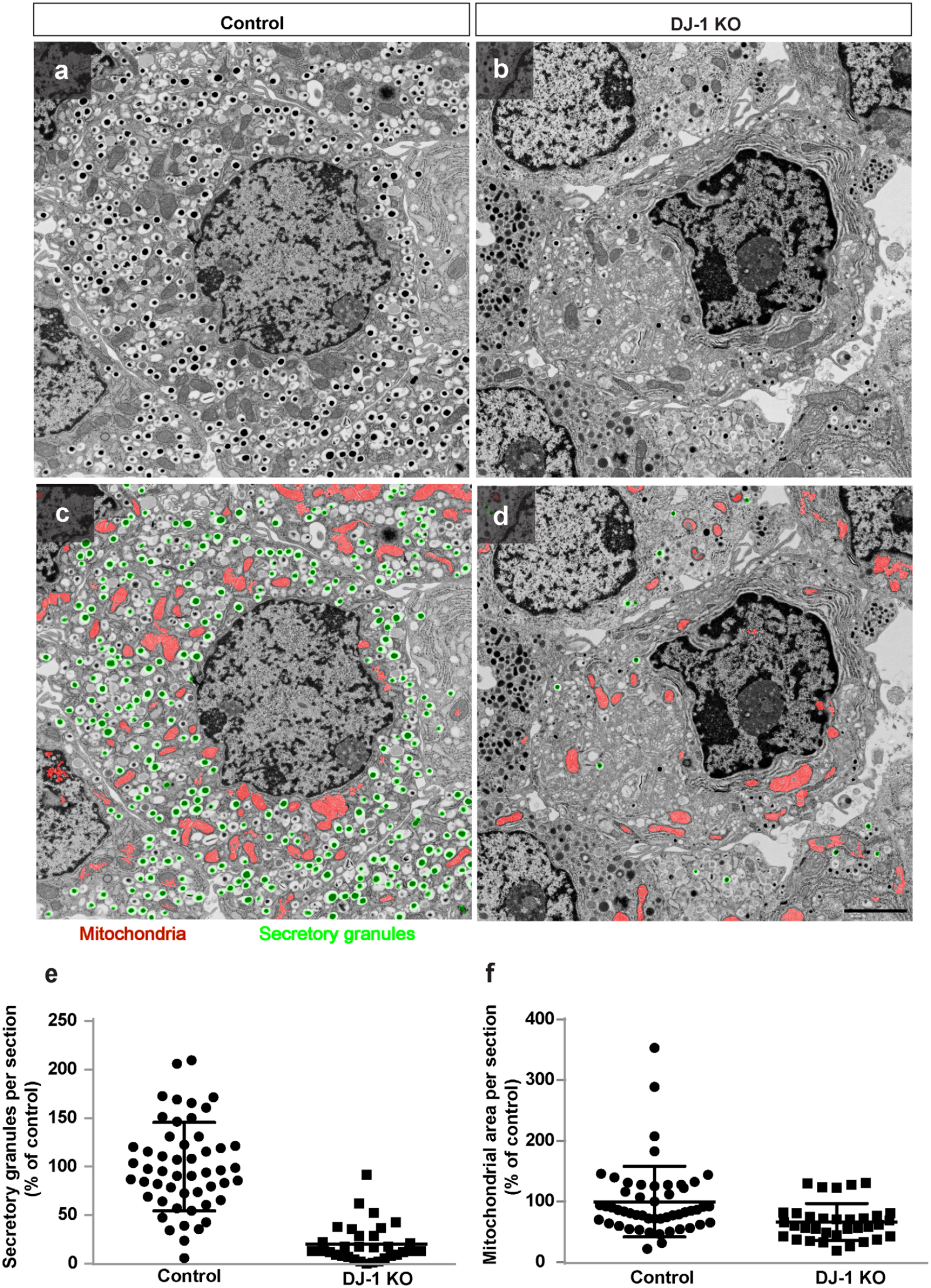
DJ-1 is required for maintaining mitochondrial morphology and number of insulin secretory granules after MLDS treatment. **(a,b)** Electron micrographs of islets from male control **(a)** and DJ-1 KO mice **(b)** after 4 weeks of MLDS treatment. **(c,d)** Image segmentation into mitochondria (red) and insulin secretory granules (green) using the trainable Weka Segmentation plugin for Fiji/ImageJ. Scale bar, 2 μm. **(e,f)** Quantification of total number of secretory granules **(e)** and mitochondrial area per section **(f)** in control and DJ-1 KO mice. n > 30 images per condition from n = 2 control and n = 3 DJ KO mice. Data are expressed as means ± SD.

In order to gain insight into the effect of MLDS treatment on different organelles of the beta cell, electron microscopy was performed on islets isolated from MLDS-treated DJ-1 KO and MLDS-treated wild type mice (Fig 4). Distinct ultrastructural abnormalities were observed in DJ-1 KO islets. More specifically, compared to islets isolated from wild type mice, the total number of insulin secretory granules and the size of the mitochondrial network were reduced (Fig 4). In sum, these data suggest that DJ-1 preserves beta cell integrity upon MLDS treatment.

### DJ-1 islet cell-autonomously contributes to the protection of beta cells from STZ-induced cell death in vitro

**Fig 5:**
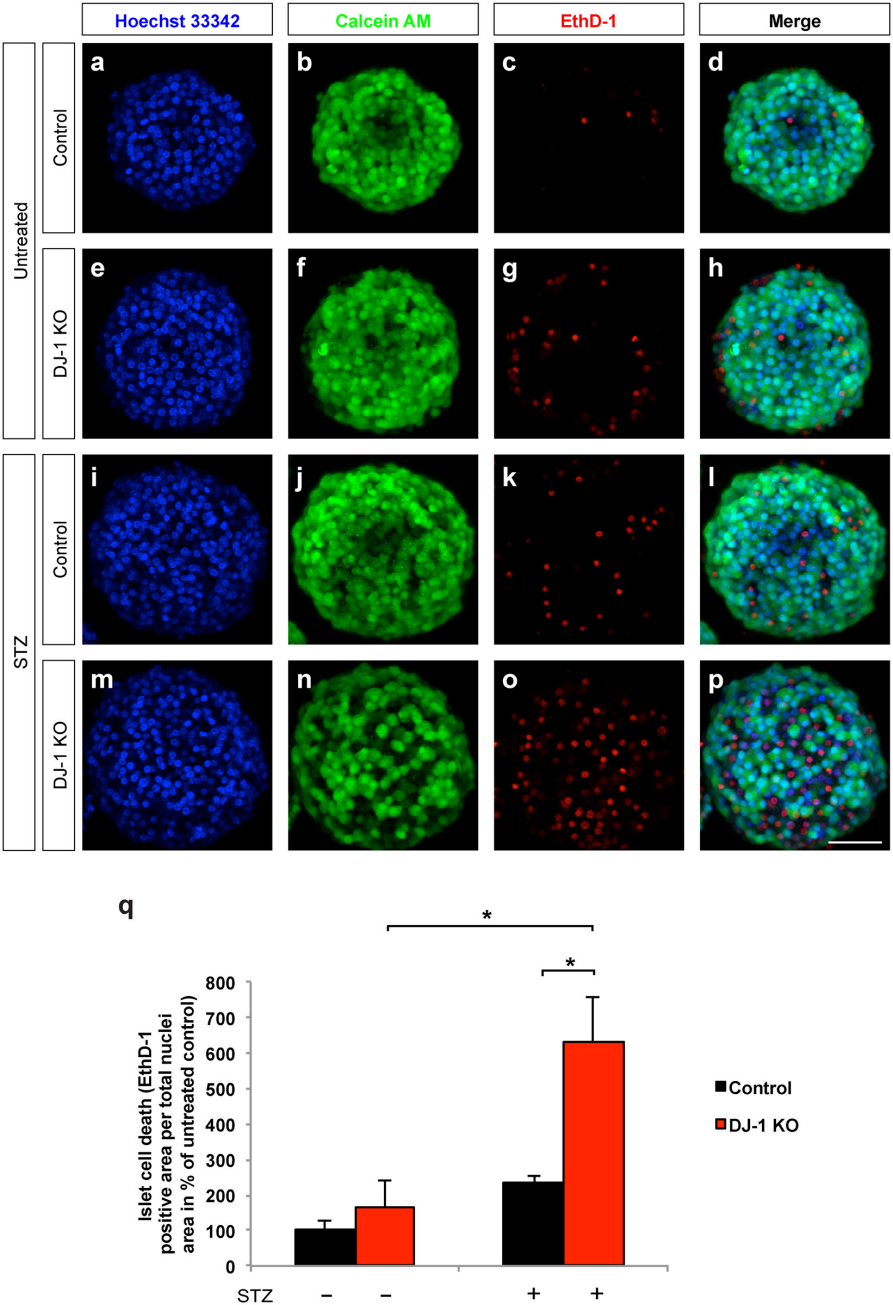
DJ-1 islet cell–autonomously protects from STZ-induced beta cell death in vitro. **(a-p)** LSM live cell images of pancreatic islets from male control **(a-d;i-l)** and DJ-1 KO mice **(e-h;m-p)** stained with Hoechst **(a,e,i** and **m)**, calcein AM **(b,f, j** and **n)**, and ethidium homodimer-1 **(c,g,k** and **o)**. Merged images are shown in **d,h,l** and **p**. Scale bar, 50 μm. **(q)** Quantification of dead cells of pancreatic islets from control and DJ-1 KO mice after 24 hours exposure to STZ treatment. Data are presented as ethidium homodimer-1 positive area per total cell nuclei area. For one experiment, islets harvested from 3–5 mice per genotype were pooled, treated and quantified. Statistical significance was assessed using the means of n = 3 independent experiments. *p<0.05 (Two-way ANOVA followed by Tukey’s multiple comparison test). Data are expressed as means ± S.D.

To evaluate whether DJ-1 expression in islets is required to protect islet cells from a STZ-induced cell death, islets were isolated from untreated DJ-1 KO and wild type mice and incubated for 24 h with 0.5 mM STZ (Fig 5). Islets were subsequently stained to detect the number of viable (calcein AM; green) and dead (ethidium homodimer-1; red) cells. STZ treatment increased islet cell death in islets isolated from wild type mice (compare Fig 5a–5d and Fig 5i–5l). Notably, upon STZ treatment DJ-1 KO islets had significantly more dead cells in comparison to STZ-treated wild type islets (compare Fig 5e–5h to Fig 5m–5p). These data show that DJ-1 is required to protect the islet cells from STZ-induced cell death.

### DJ-1 is required to reduce cytokine-mediated cytotoxicity in islets

**Fig 6:**
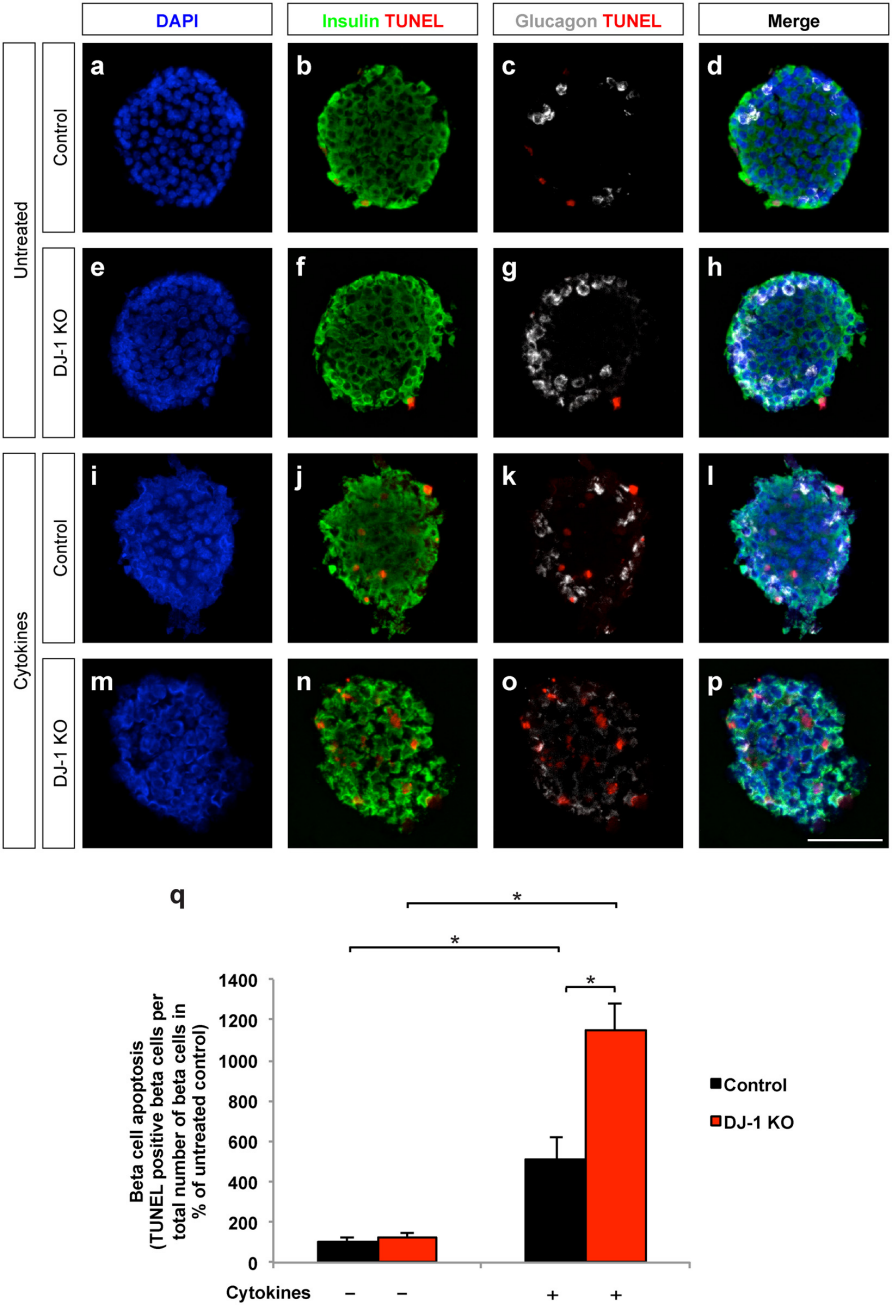
DJ-1 islet cell-autonomously protects beta cells from cytokine-induced apoptosis. **(a-p)** TUNEL labelling of pancreatic islets exposed to IL-1β, IFN-γ, and TNF-α for 24 hours. Islet sections from male control **(a-d;i-l)** and DJ-1 KO mice **(e-h;m-p)** showing staining for cell nuclei **(**DAPI**, a,e,i** and **m)**, beta cells (insulin) and dead cells (TUNEL) **(b,f,j** and **n)**, and alpha cells (glucagon) and dead cells (TUNEL) **(c,g, k** and **o)**. Merged images are shown in **d,h,l** and **p**. Apoptotic cells are shown in red. Scale bar, 50 μm. **(q)** Quantification of TUNEL positive beta cells in pancreatic islets from control and DJ-1 KO mice after 24 hours exposure to IL-1β, IFN-γ, and TNF-α. The number of apoptotic beta cells per total number of beta cells is presented as percentage of untreated control. For one experiment, islets harvested from 3–5 mice per genotype were pooled, treated and quantified. Statistical significance was assessed using the means of n = 3 independent experiments. *p<0.05 (Two-way ANOVA followed by Tukey’s multiple comparison test). Data are expressed as means ± S.D.

Cytokines released by inflammatory immune cells have been shown to contribute to increased beta cell apoptosis during diabetes progression [8, 27]. MLDS treatment was shown to result in increased cytokine production which, in part via generating reactive oxygen species, contributes to beta cell death [28, 29]. Therefore, we evaluated the role of DJ-1 during cytokine-mediated apoptosis *in vitro* (Fig 6).

For this experiment, we first isolated pancreatic islets from DJ-1 KO and wild type mice and monitored the gene expression of pro-inflammatory markers IL-1β, TNF-α and of the macrophage marker CD68 to ensure that there were no signs of inflammation in DJ-1 KO islets before treating the islets with cytokines (S3 Fig). The expression levels of the mRNA for CD68, IL-1β and TNF-α were found not to be increased in DJ-1 KO islets compared to wild type islets (S3 Fig). We went ahead and treated the islets isolated from DJ-1 KO and wild type mice for 24 h with a cytokine mix containing IL-1β, IFN-γ, and TNF-α and subsequently used them for TUNEL and insulin staining to quantify apoptosis (Fig 6).

As expected, the cytokines significantly increased the number of apoptotic beta cells in islets isolated from wild type mice (compare Fig 6a–6d and Fig 6i–6l). However, in the absence of DJ-1, the cytokines led to significantly more apoptotic beta cells compared to cytokine-treated islets isolated from wild type mice (compare Fig 6i–6l to Fig 6m–6p, Fig 6q). Thus, DJ-1 expression helps to reduce beta cell apoptosis induced by inflammatory cytokines.

## Discussion

The present study establishes that DJ-1 protects pancreatic islet cells from cytotoxic cell death, including the one induced by STZ and inflammatory cytokines. We and others could previously demonstrate that DJ-1 renders beta cells more resistant to oxidative stress and also protects and maintains the morphology and function of their mitochondria during aging and the state of insulin resistance [10, 12]. This could explain the decreased glucose tolerance in DJ-1 KO mice [10, 30], which however is not always observed [31].

Here, DJ-1 KO mice were found to be highly prone to the deleterious effect of MLDS treatment shown to induce oxidative stress. In addition, MLDS treatment not only negatively affects the morphology of mitochondria, but also reduces the number of insulin secretory granules when DJ-1 is absent from islet cells. Accordingly, upon MLDS treatment the blood glucose levels increased to diabetic conditions in the absence of DJ-1.

It is noteworthy that, although transgene expression of antioxidant enzymes such as MnSOD and catalase were shown to reduce oxidative stress in beta cells of mice, these enzymes did not confer protection against cytokine-mediated cytotoxicity in the MLDS model of diabetes [32]. Here, we now identified DJ-1 as an anti-oxidant protein involved in the protection of beta cells from cytokine-mediated cell death. Our findings are consistent with previous studies on neurons showing an anti-apoptotic role of DJ-1 under inflammatory as well as oxidative stress conditions [33].

Although it is likely that a cell-autonomous effect of DJ-1 is key to islet cell protection, we cannot rule out that under DJ-1 deficiency, and increased activity or presence of non-islet cells, such as leukocytes, contribute to the increased cell death observed under stress conditions in DJ-1 KO islets. However, we could show that Il-1β and TNF-α are unchanged and the macrophage marker CD68 is decreased rather than increased upon DJ-1 depletion before STZ or cytokine treatment.

Based on the cellular localisation and wealth of identified interaction partners of DJ-1, the protective effect of DJ-1 in beta cells can be manifold: Firstly, DJ-1 is thought to quench reactive oxygen species (ROS) [34] that can be deleterious for a cell. This may be relevant in the beta cell as ROS can be induced by cytokines and can therefore partly mediate beta cell death [28, 29]. Direct ROS quenching by DJ-1 –although limited at least in neurons [13]–can therefore potentially contribute to the protective effect of DJ-1. Secondly, DJ-1 can translocate to the nucleus upon activation and stabilize the activity of NRF2, a key regulator of the response to oxidative stress [35]. Moreover it has been shown that DJ-1 influences mitochondrial dynamics, since a mutated form of DJ-1 increases expression of mitochondrial fission protein Dynamin-like protein (DLP1) in human neuroblastoma cells, thus resulting in increased mitochondrial fragmentation [36]. A similar scenario could take place in beta cells in the absence of DJ-1 after MLDS treatment. Thirdly, DJ-1 can directly interact with proteins known to affect cell survival including proteins in the phosphatidylinositol-3-kinase (PI3K) pathway [37] and, in beta cells, TFII-I [12]. Moreover DJ-1 also influences Daxx-induced apoptosis [13]. Finally, DJ-1 reduces ER stress in beta cells [12], which is a common feature of beta cells suffering from glucolipotoxicity in T2DM subjects [38, 39]. Overexpression of DJ-1 has been shown to protect pancreatic islets from ER stress by upregulating the expression of binding immunoglobulin protein (BiP) that is important for normal function of the ER, but also part of the ER stress response [12].

Based on our findings, we propose that DJ-1 helps to protect beta cells from the negative effects of multiple stressors. Therefore, pharmacologically increasing DJ-1 protein expression or its activity using small molecules, such as the clinically used 4-phenyl butyric acid (PBA) [40] that also reduces insulin resistance in the ob/ob mouse model [41], represents a possible approach for reducing both insulin resistance and beta cell death in the future.

## Supporting Information

S1 Fig **(a)** Relative expression of DJ-1 mRNA expression in islets from male 12 weeks-old wild type (control) and DJ-1 KO mice. n = 5 mice per experimental group. *p<0.05 (Student’s t-test). Data are expressed as means ± SD. **(b-d)** DJ-1 (green, Alexa 488), insulin (red, Cy3) and DAPI (blue) co-immunostaining of wild type (**b, c**) and DJ-1 KO (**d**) islets showing the presence of DJ-1 in islets in insulin-positive beta cells, but also insulin-negative islet cells (**c**). (**b´-d´**) Same images as (**b-d**) showing DJ-1 (green, Alexa 488) and DAPI (blue) only. Scale bars for c, c´, 10 μm. All other scale bars, 20 μm.
(TIF)

S2 Fig Weight loss of male DJ-1 KO mice compared to control mice after MLDS treatment. n = 6–8 mice per experimental group. *p<0.05 (Student’s t-test with Holm-Bonferroni correction). Data are expressed as means ± SD.
(TIF)

S3 Fig Relative mRNA expression for CD68 (a), TNF-α (b) and IL-1β (c) in islets from 12 weeks-old wild type/control and DJ-1 KO mice. n = 5 mice per experimental group. *p<0.05 (Student’s t-test). Data are expressed as means ± SD.
(TIF)

S1 Data File (XLSX)
