# Supplementary figures and images for: DJ-1 Protects Pancreatic Beta Cells from Cytokine- and Streptozotocin-Mediated Cell Death
Source: PLoS One. 2015 Sep 30;10(9):e0138535. doi: 10.1371/journal.pone.0138535 (PMC4589499; doi:10.1371/journal.pone.0138535)

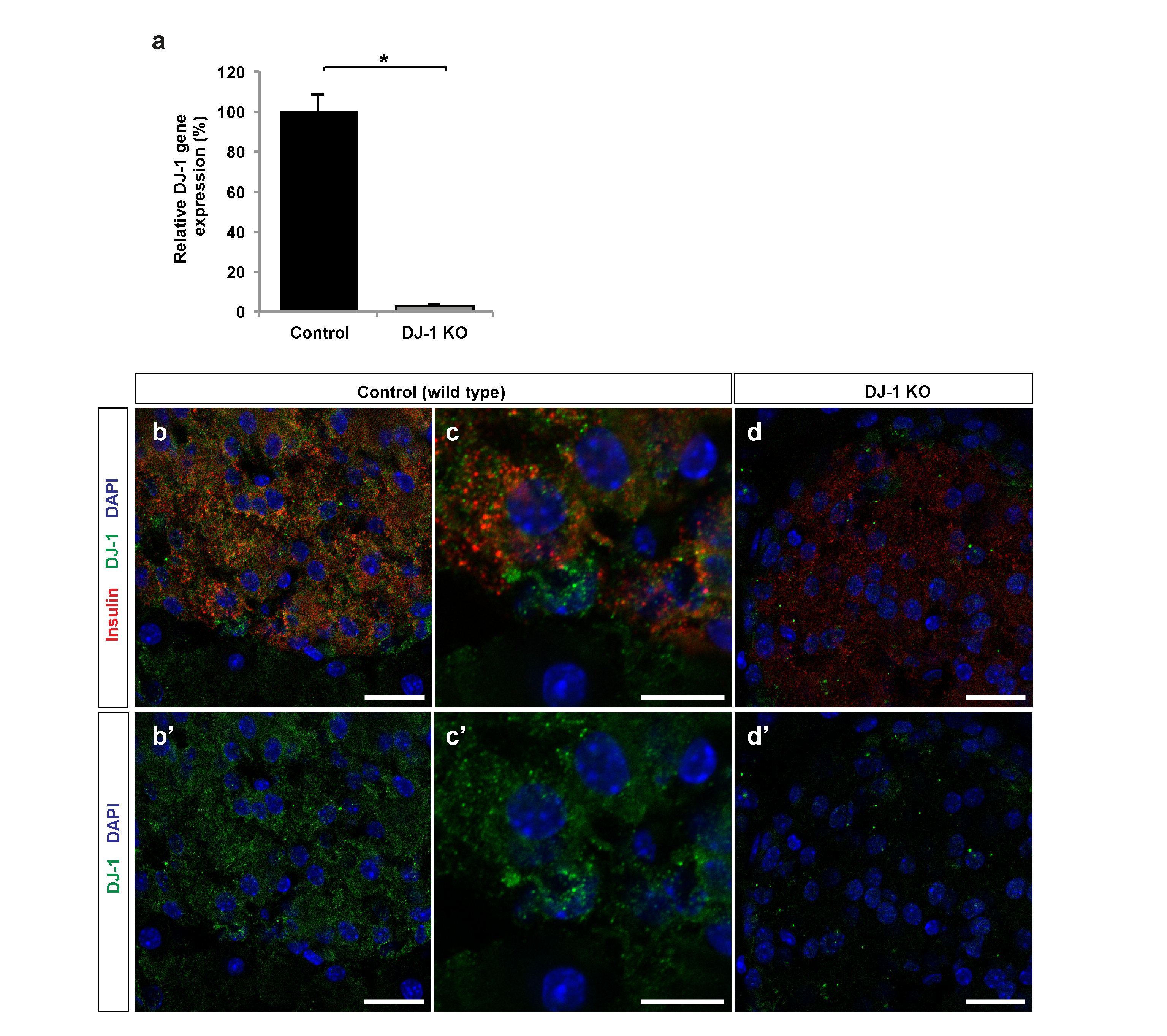

Supplement: S1 Fig — (a) Relative expression of DJ-1 mRNA expression in islets from male 12 weeks-old wild type (control) and DJ-1 KO mice. n = 5 mice per experimental group. *p<0.05 (Student’s t-test). Data are expressed as means ± SD. (b-d) DJ-1 (green, Alexa 488), insulin (red, Cy3) and DAPI (blue) co-immunostaining of wild type (b, c) and DJ-1 KO (d) islets showing the presence of DJ-1 in islets in insulin-positive beta cells, but also insulin-negative islet cells (c). (b´-d´) Same images as (b-d) showing DJ-1 (green, Alexa 488) and DAPI (blue) only. Scale bars for c, c´, 10 μm. All other scale bars, 20 μm. (TIF) [file pone.0138535.s001.tif]

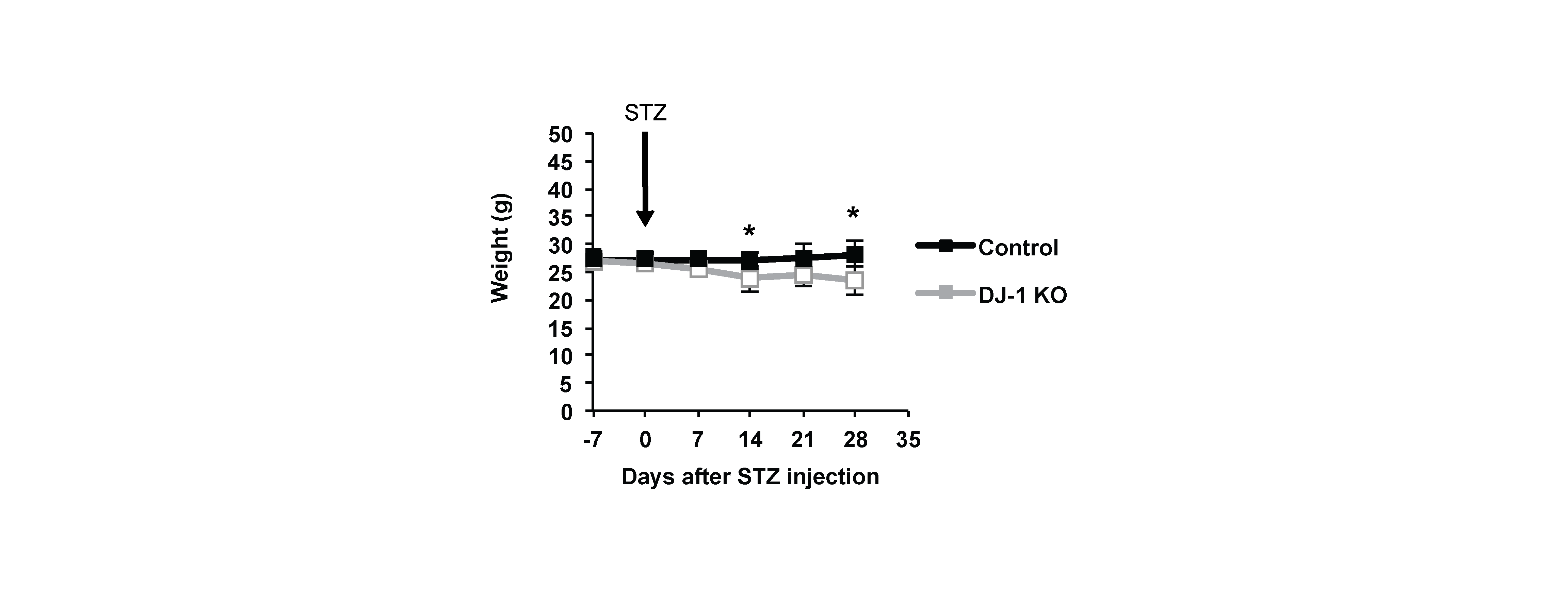

Supplement: S2 Fig — n = 6–8 mice per experimental group. *p<0.05 (Student’s t-test with Holm-Bonferroni correction). Data are expressed as means ± SD. (TIF) [file pone.0138535.s002.tif]

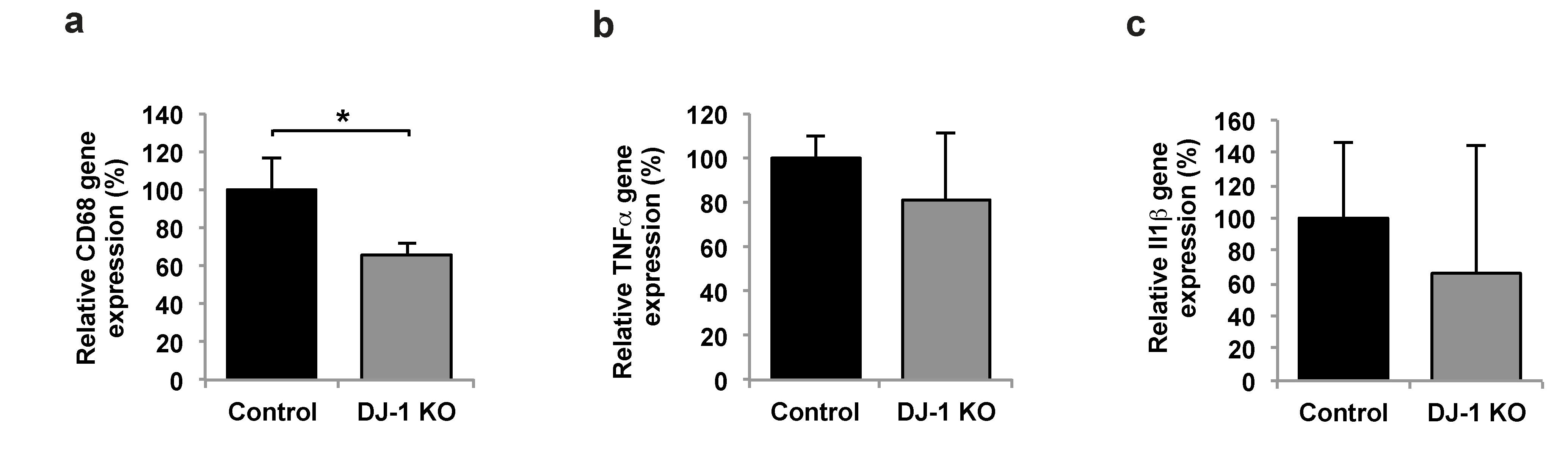

Supplement: S3 Fig — n = 5 mice per experimental group. *p<0.05 (Student’s t-test). Data are expressed as means ± SD. (TIF) [file pone.0138535.s003.tif]
